# Comparison between Arterial Blood Gases and Oxygen Reserve Index™ in a SCUBA Diver: A Case Report

**DOI:** 10.3390/healthcare11081102

**Published:** 2023-04-12

**Authors:** Fabio Di Pumpo, Gualtiero Meloni, Matteo Paganini, Danilo Cialoni, Giacomo Garetto, Alessandro Cipriano, Tommaso Antonio Giacon, Luca Martani, Enrico Camporesi, Gerardo Bosco

**Affiliations:** 1 Department of Biomedical Sciences, University of Padova, 35131 Padova, Italy; 2 ComSubIn, Italian Navy, 19025 Varignano-Le Grazie, Italy; 3 ATIP Center for Hyperbaric Medicine, 35128 Padova, Italy; 4 Emergency Medicine Unit and Emergency Department, Nuovo Santa Chiara Hospital, Azienda Ospedaliero-Universitaria of Pisa, 56126 Pisa, Italy

**Keywords:** SCUBA diving, hyperoxia, hypoxia, diving medicine, diving physiology

## Abstract

Hypoxia and hyperoxia are both worrisome issues potentially affecting SCUBA divers, but validated methods to monitor these two conditions underwater are still lacking. In this experiment, a volunteer SCUBA diver was equipped with a pulse oximeter to detect peripheral oxygen saturation (SpO_2_) and a device to monitor the oxygen reserve index (ORi™). ORi™ values were compared with arterial blood oxygen saturation (SaO_2_) and the partial pressure of oxygen (PaO_2_) obtained from the cannulated right radial artery at three steps: at rest out of water; at −15 m underwater after pedaling on a submerged bike; after resurfacing. SpO_2_ and ORi™ mirrored the changes in SaO_2_ and PaO_2_, confirming the expected hyperoxia at depth. To confirm the potential usefulness of an integrated SpO_2_ and ORi™ device, further studies are needed on a broader sample with different underwater conditions and diving techniques.

## 1. Introduction

Diving exposes the human body to several pathophysiological changes, particularly related to the increase in environmental pressure but also to breathing compressed air (or other gas mixtures), cold temperature exposure, or physical exertion, for both movement in a challenging environment and efforts to manage the diving equipment. Additionally, the diving parameters (namely, depth; diving time; and ascent speed) can influence the diving response, inducing physical and psychological stress [1].

Moreover, a peculiar adaptation mechanism—the “diving reflex”—optimizes arterial blood flow towards organs with the highest O_2_ demand [2]. Some essential mediators supporting the diving reflex’s vascular modifications are nitric oxide (NO) products catalyzed by endothelial NO synthases (eNOSs). Such mediators are fundamental in vascular flow regulation, vascular integrity preservation, and peripheral vascular tone modulation, consequently influencing arterial blood pressure [3].

The complex interplay among the abovementioned variables determines the risk of developing decompression sickness (DCS). Such diseases can affect not only divers but also astronauts, pilots, compressed air workers, and those exposed to significant variations in environmental pressure [4]. Although the occurrence of DCS per dive is relatively rare (0.01–0.1%), the prevalence of the disease is dramatically high, given the high numbers of recreational (around 11–15 million [5,6]) and professional divers around the world. Decompression is regulated by well-established procedures and translated into mandatory tables in diving planning [7]. Currently, divers can easily follow diving computer indications to mitigate the risk of DCS; however, papers recently showed that 90% of DCS happens after respecting diving computer indications, thus confirming that the mathematical approach is insufficient in preventing DCS, and a physiological, personalized approach is advised [8].

Other unique consequences of environmental pressure and gas concentration variation are hypoxia and hyperoxia, which may occur depending on the diving technique. The amount of oxygen in the sample is generally classified into three “-oxemia” categories: hypoxia (arterial partial pressure of oxygen (PaO_2_) ≤ 80 mmHg); normoxia (PaO_2_: 81–100 mmHg); and hyperoxia (PaO_2_ ≥ 100 mmHg). These three circumstances reflect different clinical conditions, such as reduced, normal availability, or excess of oxygen, respectively, in the blood and, therefore, in tissues. Although there are no unified standards, hyperoxia is roughly subdivided into mild hyperoxia (PaO_2_ 101–200 mmHg) and severe hyperoxia (PaO_2_ ≥ 200 mmHg) [9]. Several methods are available in clinical practice to analyze the oxygenation state. Arterial blood gas (ABG) analysis is the most reliable test for analyzing oxygen content in arterial blood. Another valid parameter is arterial oxygen saturation (SaO_2_) or the amount of O_2_ carried by the hemoglobin in arterial blood, expressed in terms of percentage, which is still measured in arterial blood. The most non-invasive method used is pulse oximetry, especially to detect and monitor for hypoxia by tracking peripheral oxygen saturation (SpO_2_) [10,11,12]. In healthy people resting and breathing at sea level, a value above 95% is considered normal [13]. However, pulse oximetry is not useful to inform on the status of an acute excess of PaO_2_ (more than 100 mmHg) because the maximum SpO_2_ value is 100%. During intubation in the operating room, or during emergency airway management in the emergency department or in the prehospital arena, pre-oxygenation of the patient provides a significant reserve to both the elective and critically ill patient [14]. Other than protective pre-oxygenation, hyperoxia can be inadvertently attained, for example, in intensive care or anesthesia, and be harmful for patients. To measure such excess, the oxygen reserve index (ORi™) has been developed and recently consolidated [9,15,16,17]. ORi™ is expressed through a dimensional index (with scores between 0 and 1) estimating PaO_2_ from approximately 100 to 200 mmHg, representing mild hyperoxemia [15]. This index has been developed to prevent the well-known consequences of unnecessary oxygen administration, both acute (such as resorption atelectasis) or after prolonged exposures (thus inducing multifaceted and probably harming effects) [17], but is not useful in measuring hypoxia.

Blood oxygenation monitoring could be useful to prevent accidents in diving. Underwater hypoxia is potentially fatal for breath-hold divers or closed-circuit rebreathers (CCR), quickly leading to a sudden loss of consciousness and drowning without warning symptoms [1]. Acute hypoxia can present with different symptoms, such as tremors, difficulty concentrating, confusion, memory loss, visual impairment, anxiety, depression, euphoria, dyspnea, paresthesia, headache, dizziness, tachycardia, and a loss of consciousness. In addition, severity and velocity in the onset of hypoxia symptoms widely vary across individuals, hence its difficult predictability. In breath-hold divers, hypoxia usually manifests while ascending and results from the interplay of oxygen metabolic consumption and gas redistribution throughout the body and the re-expanding lung, in some cases leading to loss of consciousness [18]. Hypoxemia at depth and after surfacing has previously been demonstrated through arterial blood gases on breath-hold divers [19,20,21]. As a continuous monitoring or screening tool, SpO_2_ is more feasible given its non-invasive nature in breath-hold diving and CCR. SpO_2_ can also be a valuable indicator of diving-induced pulmonary edema: a delay in re-achieving 95% greater than 20 min after surfacing can suggest the development of pulmonary acute disease [22,23].

On the opposite side, hyperoxia during diving can cause seizures and lead to fatal events but has few warning symptoms. Transient hyperoxemic states are usually encountered at depth during breath-hold diving by most subjects, as protection, ensuring sufficient time to reach the planned depth and to resurface [20]. Hyperoxia is also attained in SCUBA divers [24] or in divers using CCR. Given its measurement limits, pulse oximetry does not inform on hyperoxemia levels—as SpO_2_ reaches 100%—and ABGs are not feasible as well for common users; instead, ORi™ could be the only available technology suitable for underwater hyperoxemia monitoring.

It is, therefore, evident how SpO_2_ and ORi™ measurements could be useful underwater to detect oxygen derangements on both sides of the curve: SpO_2_ could help in detecting hypoxemia, while ORi™ could quantify a hyperoxemic condition. While SpO_2_ underwater is already available through marinized finger pulse oximetry, such a configuration for ORi™ has still not been developed and tested. In this paper, a new device was designed to insulate ORi™ and test it for reliability on a SCUBA diver underwater, comparing it with arterial blood gases.

## 2. Materials and Methods

The volunteer for this study was a 31-year-old, non-professional SCUBA (open-circuit) diver with the following characteristics: weight, 75 kg; height, 180 cm; non-smoker; no diseases reported at past medical history; obtains an annual medical evaluation for recreational diving suitability as per Italian sports activities recommendations. SCUBA (open-circuit) diving was chosen as the first technique to test the new apparatus as the most feasible in the experimental setting. The experiments were performed at the Y-40 swimming pool in Montegrotto Terme (Italy) to conduct pilot testing in controlled conditions. After obtaining informed consent, the subject underwent a physical examination by one physician on site, which was unremarkable.

A Masimo Radical-7 pulse oximeter (Masimo Corporation, Irvine, CA, USA) was sealed in a waterproof container connected to a finger sensor inside a standard diving dry glove. This setup was chosen to avoid the interposition of water between the sensor and the finger, potentially altering light refraction and measurements. For ORi™ (ver. 1.6.2.4 i-dm), an RD rainbow SET^TM^-2 (Masimo Corporation, Irvine, CA, USA; rev. 0) was used inside a standard diving glove, as above.

For arterial blood gas sampling, the same technique used by Bosco et al. in previous studies [20,21] was adopted. Briefly, on the non-dominant limb, an Allen test was performed to verify adequate hand perfusion. After local anesthesia, an arterial cannula was inserted into the radial artery. The arterial catheter was fixed to the skin using an adhesive band and then connected to a circuit with Luer Lock-type connectors to prevent leakage. The circuit was composed (in a proximal–distal order) of one rigid plastic connection, single lumen, 10 cm in length; one lockable three-way stopcock; one 2.5 mL heparinized plastic syringe for standard blood gas sampling on the first stopcock port; one 10 mL plastic syringe filled with 3 mL of 0.9% NaCl solution on the second stopcock port, used both for arterial blood aspiration before sampling (“dead space”) and for flushing and washing out the arterial line after the sample collection. The whole circuit was filled with 0.9% NaCl solution, ensuring that any gas bubbles had been removed. In the case of disconnection, subjects were trained to turn the stopcock or apply direct pressure to prevent any bleeding. Blood collection underwater consisted of rotation of the stopcock; aspiration of 5 mL of blood (dead space); rotation of the stopcock towards the heparinized syringe; aspiration of arterial blood; rotation of the stopcock back to the first syringe; flush of the circuit; positioning of the stopcock in an intermediate position to close all channels.

**Figure 1:**
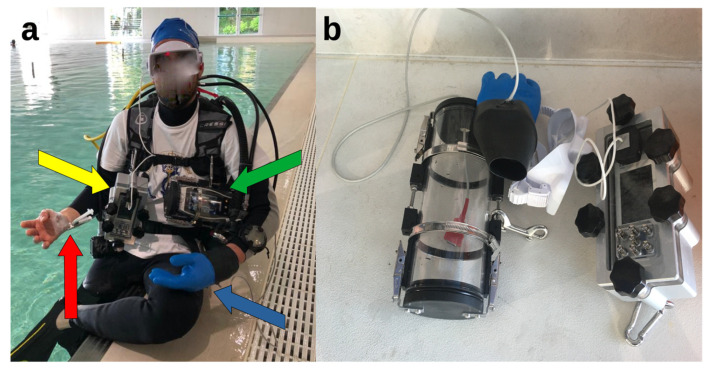
The experimental set-up at the poolside. (**a**) The volunteer SCUBA diver equipped with marinized pulse oximeter (yellow arrow) and ORi™ (green arrow) devices; the arterial access on the non-dominant hand (red arrow); the SCUBA diving glove (blue arrow). (**b**) Insulating devices for the pulse oximeter (on the right) and for the ORi™ device (on the left, with its sensor inside the SCUBA diving glove).

At the end of the experiment, the arterial cannula was aseptically removed, and a compression bandage was applied for two hours. The insertion area was monitored over the next two days for complications. The complete set-up for the experiment and the insulating devices are shown in Figure 1.

**Figure 2:**
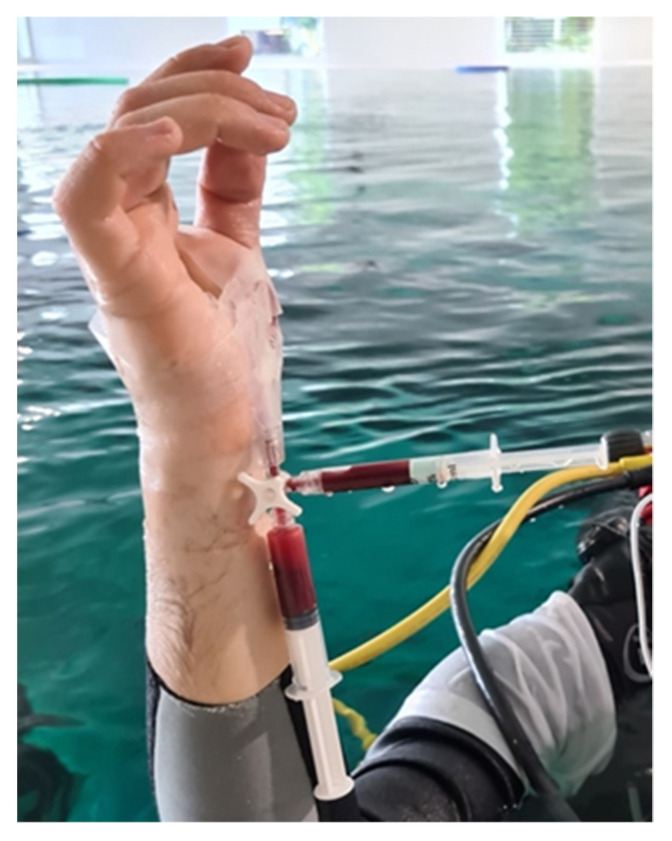
Third arterial blood sample at the surface. The heparinized syringe (pointing rightward) was analyzed to compare partial pressure of oxygen with ORi™ values. The syringe pointing downward was only used to draw initial blood and then flush the line (dead space syringe) and was, therefore, discarded.

Blood gas analysis, pulse oximetry, and ORi™ were obtained at the following subsequent steps:

1. At the surface, on the stretcher after the arterial cannula placement, with the diver breathing ambient air (dry conditions, rest);
2. Underwater, at a depth of −15 m of free water (mfw), after 15 min spent on an underwater bicycle (OKEO, Genova, Italy) pedaling at a rate of 25 rpm to ensure no difference in ventilation and gas exchange in all dives, guided by the Borg category ratio 0–10 scale at an intensity of 3 [25], with the SCUBA diver breathing compressed air;
3. Immediately after resurfacing from the dive, head out of the water, with the diver still breathing air from the SCUBA apparatus (Figure 2).

Arterial blood samples were limited to three to avoid excessive discomfort to the volunteer and analyzed within one minute using an analyzer present on site (Stat179 Profile Prime Plus, Nova Biomedica Italia Srl, Lainate, Italy). SaO_2_ and PaO_2_ were measured and compared to the ORi™ values in the same three steps. In addition, the data from the pulse oximeter and ORi™ registered throughout the dive were also visually analyzed to check for minimum and maximum values.

All the procedures were carried out in accordance with the Declaration of Helsinki and approved by the institutional ethics committee.

## 3. Results

Both the SpO_2_ and the ORi™ showed a constant trend throughout the dive. The SpO_2_ profile did not show hypoxic events, with a maximum registered value of 100% (bottom) and a minimum of 95% (beginning). ORi™ measured a maximum of 1 (bottom, while pedaling) and a minimum of 0 (at the beginning).

Compared to the three arterial blood samples, SpO_2_ and ORi™ grew from the surface to the bottom and then returned to pre-dive values. In particular, ORi™ demonstrated hyperoxia at the bottom after pedaling (0.23), consistently with the PaO_2_ measured at the same time (236.7 mmHg), while both SpO_2_ and SaO_2_ reached the predicted 100%. After resurfacing, oxygen levels in all the domains returned to normality, as ORi™ dropped back to 0, demonstrating a non-hyperoxic state (Table 1).

## 4. Discussion

This is the first study to specifically compare arterial blood gases SaO_2_ and PaO_2_ with ORi™ in a SCUBA diver while testing the feasibility of such a monitoring setup underwater.

No malfunctioning was reported after the experiments, and the devices could record the intended parameters at any moment. Such a configuration, still at an early stage, could be improved by including several other devices in a compact, wearable, and commercially available apparatus. Current developments in monitoring technology, as well as recent developments in telemedicine, are a spur to find the best configuration for constantly monitoring divers’ activities and health status [26].

The data collected by the pulse oximeter and the ORi™ mirrored the arterial oxygen variations during the dive, especially demonstrating hyperoxemia for the bottom sample after pedaling (PaO_2_ = 236.7 mmHg; SaO_2_ = 100%), high peripheral oxygen saturation (SpO_2_ = 100%), and hyperoxia at ORi™ (0.23).

It should be noted that the ORi™ may have inherent limits in underwater measurements due to the identified hyperoxia range. As per the manufacturer’s specification, ORi™ can identify a moderate hyperoxia range or PaO_2_ between 100 and 200 mmHg. In immersion with air, as the depth increases, the partial pressure of oxygen (PO_2_) increases, becoming toxic at an M.O.D. (maximum operative depth) of −57 m (1):

M.O.D. = (PO_2_/FO_2_ − 1) × 10 = (1.4/0.208 − 1) × 10 = 57 m (1)

Even at irrelevant depths, such as the depth of this experiment, breathing compressed air can result in hyperoxia: at −15 mfw, the PO_2_ is depth × FiO_2_ = 2.5 atm × 0.21 = 0.52 atm. In a dive with air, this aspect could lead to slight hyperoxia for the entire dive duration. In this experiment, increased ORi™ values confirmed slight hyperoxia development without hyperoxic problems. At another moment, not compared with arterial blood gas, ORi™ reached a maximum of 1 (shortly after the underwater arterial sampling) and then normalized immediately, at the surface, without any symptoms. A similar trend can be visualized in the PaO_2_ of the three blood samples, where the value was normal in the first sample (103.3 mmHg), increased in the bottom sample (236.7 mmHg), and returned to near normality on the surface, despite being slightly above the threshold due to the chest still being underwater (108.3 mmHg).

Effectively, the ORi™, as proposed, could suggest an initial state of hyperoxygenation while diving without necessarily corresponding to overt symptoms or real dangerous situations and, therefore, warn the diver to avoid hyperoxemia as a carrier of harmful redox imbalance toward oxidative stress, even at mild levels. In fact, exposure to extreme environmental conditions increases stress hormones and inflammatory biomarkers [27,28,29]. When the human body is exposed to changes in environmental pressure or oxygen concentration levels, alone or in combination, many adaptions occur, depending on the specific condition and activity performed [27,30]. There is a clear link between activities performed at pressure and acutely increased oxidative stress due to the rise in the arterial partial pressure of oxygen and, therefore, higher oxygen concentrations in tissues [29,30,31,32]. Physical activity also induces oxidative stress due to increased oxygen requests to cope with muscle demands, resulting in reactive oxygen species (ROS) production acting as signaling molecules but also influencing performance [33]. The antioxidant response is an adaptation that occurs to cope with potential damage induced by oxidants. It is enhanced under chronic conditions in, for example, adequately trained athletes; instead, under acute exposure, our body is not ready to cope with increased oxidative stress [34,35,36]. Such aspects have been limitedly investigated in breath-hold diving [31,37]. In SCUBA diving, divers exposed to hyperbaric and hyperbaric hyperoxic conditions showed increased DNA damage and oxidative stress markers, which is more pronounced with hyperoxic air mixtures [38]. The ultimate effects of redox alterations induced by this particular condition have not been fully explored yet [39]. Subjects involved in professional and industrial diving usually are exposed to hyperbaric conditions for weeks–months continuously into saturation implants. These conditions increase oxidative stress markers, antioxidant responses, and DNA damage [29,40,41].

With this perspective, ORi™ could act as a preventive monitoring device to alert the diver to hyperoxemic states—prompting safety initiatives such as dive interruptions or mixture variations during technical dives—and avoid excessively long or repetitive dives in order to prevent oxidative damage. The potential applications of such monitoring devices could cover different diving techniques, especially those exposing subjects to prolonged oxygen concentrations or high pressures. For example, technical divers, using rebreathers or mixtures to prolong their stay underwater at deep depths, could routinely wear hypoxia- and hyperoxia-monitoring devices to guide their diving profiles and choose to extend or shorten their explorations while staying below an oxidative stress threshold—a concept still to be elaborated in environmental physiology. Additionally, for saturation divers, this hypoxia–hyperoxia monitoring could have several occupational medicine implications to limit professional divers’ exposures and improve their health. If further studies confirm the ORi™ reliability, the device’s measurement range should be extended to enhance hyperoxia detection and accuracy.

ORi™ measurements can be influenced by several factors, such as movements of the body or low-perfusion states [42]. Additionally, given the similar PaO_2_, different values in ORi™ can be obtained from different subjects [42]. With this perspective, it is reasonable to understand how the 0.23 value obtained at depth could have been influenced by both movements of the body or perfusion, remembering that the diving reflex causes blood redistribution from the peripheral circulation to the chest. This is a spur to perform further experiments on other subjects and, potentially, to work directly on the software or the probe to improve measurements, taking into account the peculiarity of underwater pathophysiological adaptations. Another limitation of this report, apart from the values being obtained from a single volunteer and a single dive, includes the warm temperature of the water; experiments in open waters could lead to different conclusions and should be investigated.

## 5. Conclusions

Integrated oxygenation telemonitoring while diving could improve diving safety in the future. While pulse oximetry could be useful in breath-hold and CCR divers to detect hypoxia, ORi™ seems a promising monitoring device for the early detection of hyperoxemia, potentially exposing SCUBA and CCR divers to the risk of acute oxygen toxicity underwater and harmful oxidative stress.

This experiment can be considered the basis for subsequent studies evaluating the feasibility of such monitoring in a broader sample of volunteers in open waters and with different diving techniques, ranging from breath-hold divers to CCR and technical diving with mixtures. Integrated data from SpO_2_ and ORi™, in the future, could become the most feasible and reliable monitoring parameters to avoid hypoxia and hyperoxia, which are events that frequently happen in CCR divers.

## Figures and Tables

**Table 1:** Comparison between arterial blood gases (ABGs) and peripheral oxygen saturation (SpO_2_) measured through a pulse oximeter and the oxygen reserve index (ORi™) during three different steps.

Event	Time	ORi™	SpO_2_ (%)	Heart Rate (bpm)	Dive Time	Depth (mfw)	Water Temperature	pH	PaCO_2_ (mmHg)	PaO_2_ (mmHg)	SaO_2_ (%)
ABG at rest, out of water	17:49:00	0.0	98.0	80.0	-	-	(Out of water)	7.398	38.7	103.3	98
ABG at 15 mfw	18:31:01	0.23	100.0	108.0	18:00	13.5	Not available	7.339	47.4	236.7	100
ABG—end of the dive	18:37:41	0.0	96.0	106.0	24:40	2.5	35°	7.418	36.9	108.3	98

## Data Availability

Data are available from the authors upon reasonable request.
